# An Account of the North Kensington Friendly Workers among the Poor

**Published:** 1892-09-17

**Authors:** C. Furley-Smith


### THE CURE OF POVERTY,

### AN ACCOUNT OF THE NORTH KENSINGTON FRIENDLY WORKERS AMONG THE POOR

—(*continued from page* 400).

By MRS. C. FURLEY SMITH.

IN first forming the Association it was agreed that help, whenever given, was to be ample and sufficient; that it must be so if the effect was to be permanently beneficial. Where there was a fair possibility of ultimate repayment money was given as a loan; but not otherwise. A gift was never called a loan, it being felt that so to misname it was only to encourage a breach of faith. But in several cases loans or gifts were sanctioned, so large as probably to surprise—perhaps even shock—the average donor, but always bestowed with discretion, and successful in the object for which they were intended, obviating the necessity for further help. The case which has been already quoted was an instance of this. The five pounds given by the Campden charities made it possible for the Committee to feed three children properly and make such arrangements for taking care of them as set the father free to look for and obtain work, thereby setting the whole family on a solid basis of comfort and well-being for the future. But of course sovereigns and five-pound notes cannot be thrown about promiscuously; enough mischief is done by the too careless distribution of shillings. These gifts were guided and justified by that personal investigation of, and acquaintance with, the circumstances of each case, which was one of the Association's fundamental laws. Alms-giving alone was never its object; it was employed only as a means to independence, and its form and amount was always adapted as carefully as might be to that end.

Very little in the way of "relief works" was attempted. That, at best, is a temporary expedient, which must one day be discontinued, and, therefore, was unsuitable to the special aim of the Association, viz., the elevation of the poor into a condition of independence. But work was found for some job bootmakers in this way. Clothing of any kind was a valuable help to the people by the Association, but there was a difficulty about the article most serious for the poor to buy—shoes. Anyone who has watched the faces of poor women looking in at the windows of those establishments where the repairs of boots and shoes are done in presence of the public will have noticed the strange and eager interest they display. As a matter of fact, they are studying, trying to pick up hints as to means and methods which will enable them to mend their children's boots at home. I know one woman, well-to-do in her way, but frugal as John Gilpin's wife, who has procured a set of cobbler's tools, and mends and patches in a way wonderful to behold. But with all this, the question of shoes remains one of the most insoluble to the poor. New boots make, even in the most prosperous times, a big hole in a week's wages; in times which are unprosperous they are impossible luxuries. And the difficulty is that boots are very rarely given away. Women may send off their hats and gowns when they get faded and out of fashion: men will part with their suits when they are shiny at the seams; underclothing goes when it is in need of persistent mending; but no one parts with boots until they cease to be watertight. But to people who are not fastidious about patching and re-soling, they may be made wearable. Thus the Association found it practicable to take boots in even the most dilapidated condition, and have them mended by some of the cobblers they knew, and for whom they wished to find work, before selling them to the poor of the district.

Selling—not giving. This was the rule, occasionally departed from in special circumstances, but in the main adhered to. Clothes given for nothing are apt to find their way to the pawnshop; therefore, though the clothes collected from the well-to-do people of North Kensington for their poorer neighbours were disposed of for less than their value, they were always sold for more than a pawnbroker would give for them. People who have found out how gifts of clothing are wasted will appreciate the value of this method. In another way it was contemplated to help the poor in the matter of clothes. This was by the establishment of a workroom under the following rules:—

1. The workroom to be (1) primarily for the instruction of women in making, darning, patching, cutting out, knitting stockings, &c.; (2) secondly for the instruction of such of the women as are willing to be taught "fine" needlework; and (3) thirdly to provide help for competent workwomen who are out of employment and have none but their own mending work to attend to at home.

2. Only women of good character, who are temporarily out of work or whose work is temporarily insufficient, and who have been at least a year in the district, to be employed without the special sanction of the Committee on the written representation of the Superintendent.

3. No woman to be employed more than four weeks at a time, or for more than thirteen weeks in the year.

4. No work to be given out.

5. No clothes for sale to be made, but women to be allowed to bring their own garments, or those of members of their family, and make or mend them, in lieu of working on the material provided in the workroom for instruction.

6. The workroom to be opened every week day from one p.m. to five p.m.

7. Any person or society recommending a worker, to pay on her behalf to the Superintendent the sum of 1s. 2d. for every half-day's work. One shilling to be paid by the Superintendent to the worker, less a fine of 1½d. for every half-hour absent, the remaining 2d. to be retained for the expenses of the workroom.

As a matter of fact, the work-room was never established because it was found practicable, where help of this kind was needed, to make use of the workrooms belonging to some of the churches in the district. But as many of the rules of these were similar to those formulated for the Friendly Workers, we have thought it advisable to give the latter here.

So much for detail of method: in conclusion, it is right to give a few details of result. As we have already said, the aim of the Association was to help the poor to become self-supporting. But in every district one finds people whose working days are done, and who have been able to save little for old age. For such as have only their own improvidence to blame for this the parochial authorities are the fitting helpers; but there are many decent folks who deserve some better fate than to die in the workhouse. If they are poor it is not their fault, and one would fain see their last days made comfortable. Take for example such a case as the following:—

Case of an old woman, aged 70 (single, who had left service six years, where she had saved a fair sum of money, on which she had been since living. At time first visited she had £12 of her savings only left, and we were asked by the visitor to try and get some friends to take an interest in her and give so much a week to pay her rent. She had no relations who could help, her three brothers and two of their wives being dead. She had been in service over fifty years, and had lived a long time in the neighbourhood. The case was taken up, and through the kindness of a gentleman in whose family she had been servant, and that of a lady interested in our Society, the sum of 2s. 6d. a week was paid till within a month of her death, when her needs were attended to by St. Columb's Church.

It certainly would have been cause for regret if this old woman, who had kept herself so long, had been forced to give up her self-respect in her latter days, to the point of going into the Union. Having done her best to help herself, she deserved help from others. Probably if she had met with something like the Friendly Workers when she first left service, she would have been advised and helped to buy an annuity with her savings, or part of them, instead of eating into her capital. This old woman needed, and deserved, help to keep a roof over her head in her last days. But actual alms were not always required; only a wise head and a friendly hand to help honest people out of difficulties. Take this case:—

Case of a cabman who had formerly lived in the North of England, where he had belonged to an Oddfellows Club. He had always continued to pay his subscriptions, but through what appeared to us to be dishonesty on the part of the club, he has not been able when ill to get any money paid him. We took the case up, and after some weeks of correspondence with various officials of the club, whom we threatened to prosecute if the money was not paid, and with the assistance of the Post Office authorities in tracing the man's payments, we got him the money due to him.

Here an honest and provident man was being wronged, and would probably have been cheated out of his money if the Association had not taken up the matter. In such cases as this the advantage of having as the agent of the Friendly Workers an educated man who knows the world is obvious. The club was evidently trusting to the cabman's ignorance and inability to prove his continued payments, and might have succeeded in cheating him if there had been no one to take the case up and carry it through.

It is this intelligence and common sense that are so much needed in dealing with the poor. They fall into hopeless difficulties through sheer ignorance of what to do in cases in which other people consult their solicitor. Here were a mother and son who really wanted to do well in the world, but lost through a bad tenant:—

Case of a woman and son who rented a house in a poor street and sub-let. Complaint made that the family in kitchen floor were most disorderly, and ruined the prospect of the house. We visited, and went into the question, and found that in kitchen floor a woman was living with her two children, and that the same room was often also used at night by an unmarried couple. We also visited this woman, and told her that unless she kept her floor respectably, we should take steps to have her removed, and tried by kindness to persuade her to give up her past ways, and offered if she would do this to help her make herself and children more comfortable. All was, however, of no avail, and she had to be got away. The condition of the house had caused the woman who had rented it to lose considerably, and she herself was unable to any longer pay her way there. Her son was a job bootmaker, and we have since aided him in setting up a shop, where he is doing fairly well, and his mother lives there with him. The case has also been helped, in conjunction with ourselves, by the Roman Catholic clergy.

A similar case, in which a woman had injured herself by too great kindness to others, may here be quoted:—

Case of married woman, whose husband had been out of work some time (slipper making).—They had formerly rented a house and sublet, but owing to laxity through kindness, in collecting rents, they lost all the money they had, and when we found them, the woman had pawned nearly everything she had possessed, including her own and husband's clothing, bed-clothing, &c. We took the case up, and found the wife had been in much better circumstances, and had borne a good character, and that her distress was really caused more through unwillingness to be hard on others than from any fault of her own. We were able to collect (to a large extent through the kindness of a lady, with whom the woman's father had been a farm servant), sufficient money to redeem a great many of the most necessary articles pawned, and we have now every reason to believe, as the husband is now in work and they have no longer the house on their hands, that for the future they will be able to have a comfortable and happy home.

In these cases the chief tenants of the respective houses showed themselves distinctly unbusinesslike, and suffered through their lack of business capacity. The loss was inevitable in their station as much as in any other; but with them, as with others, it was a good thing that there was a friend to prevent loss degenerating into irretrievable ruin. To let people drift into the class of hopeless paupers because they have made one foolish speculation is stupid as well as regrettable. Of course, there will always be cases which, if dealt on in the severest spirit of political economy, would not be helped at all. Here is one:—

Case of a married couple where the husband is very much older than the wife, and cannot be got to do any work, whilst the woman is an example of persevering industriousness. We have tried all we could to induce the husband to go to the Workhouse Infirmary as he is very often ill, but find it quite impossible to persuade him to do so. Under the circumstances, we have done all we can to get the wife work, and in this matter have been most successful, which has especially resulted from the careful and thorough manner in which she has always carried out any work entrusted to her. She has been kept pretty regularly at charing work, and during the summer has had the charge of an empty house during the absence of the family. The one unsatisfactory part has been, and still is, the fact that the woman has, out of her earnings, to support her husband, who ought to be in the infirmary if too ill to work.

In this case one would rather help the wife alone than the wife as supporter of the husband. But as the Lord of the Harvest has ordained that wheat and tares shall grow together until the harvest, we must be prepared for the tares occasionally getting the benefit of that which was meant only for the wheat.

*(To be continued.)*

